# Supplementary material for: Medial temporal lobe functional connectivity predicts stimulation-induced theta power
Source: Nat Commun. 2018 Oct 25;9:4437. doi: 10.1038/s41467-018-06876-w (PMC6202342; doi:10.1038/s41467-018-06876-w)
Supplement: Supplementary file 1 — Supplementary Information [file 41467_2018_6876_MOESM1_ESM.pdf]

Medial temporal lobe functional connectivity predicts stimulation-induced theta power

Solomon, et al.

Supplementary Information (2 tables, 4 figures)

Subject ID	Z	P	Region	Distance from WM
**1096**	3.016455	0	left ca1	0.989909
**1101**	3.411592	0.001	left fusiform gyrus wm	1
**1113**	1.72762	0.044	left sub	0.99386
**1114**	3.606953	0	left mtl wm	1
**1114**	1.659178	0.041	left amy	0.950779
**1115**	1.627038	0.045	left sub	0.99555
**1120**	3.919829	0	left mtl wm	1
**1122**	2.604052	0.003	right sub	0.995395
**1125**	1.737326	0.043	left prc	0.988886
**1125**	1.750235	0.027	left prc	0.981156
**1125**	3.090451	0.005	left phc	0.997158
**1134**	1.877686	0.034	left prc	0.994108
**1144**	1.647388	0.039	left phc	0.994771
**1144**	1.695835	0.048	left sub	0.995736
**1144**	2.851826	0.014	left sub	0.994648
**1153**	2.043864	0.021	left phc	0.995562
**1163**	3.423	0	left prc	0.990563

Supplementary Table 1. Stimulation sites with significant ($P < 0.05$) theta modulation index. P-value were calculated relative to a null distribution; 0 indicates the true correlation exceeded all 1000 observed null correlations. Distances are reported as $e^{-(dist)}$, where 1 indicates electrodes placed in white matter. Legend: WM, white matter; sub, subiculum; amy, amygdala; prc, perirhinal cortex; phc, parahippocampal cortex. “mtl wm” refers to any white matter in the parahippocampal gyrus.

Stimulation region	Count
**left amy**	1
**left ca1**	10
**left dg**	3
**left ec**	2
**left phc**	5
**left prc**	12
**left sub**	6
**left mtl wm**	5
**right amy**	1
**right ca1**	13
**right ca2**	1
**right dg**	3
**right ec**	2
**right prc**	5
**right sub**	1
**right mtl wm**	2

Supplementary Table 2. Count of stimulation sites for each MTL subregion. Abbreviations are listed in legend for Supplementary Table 1.

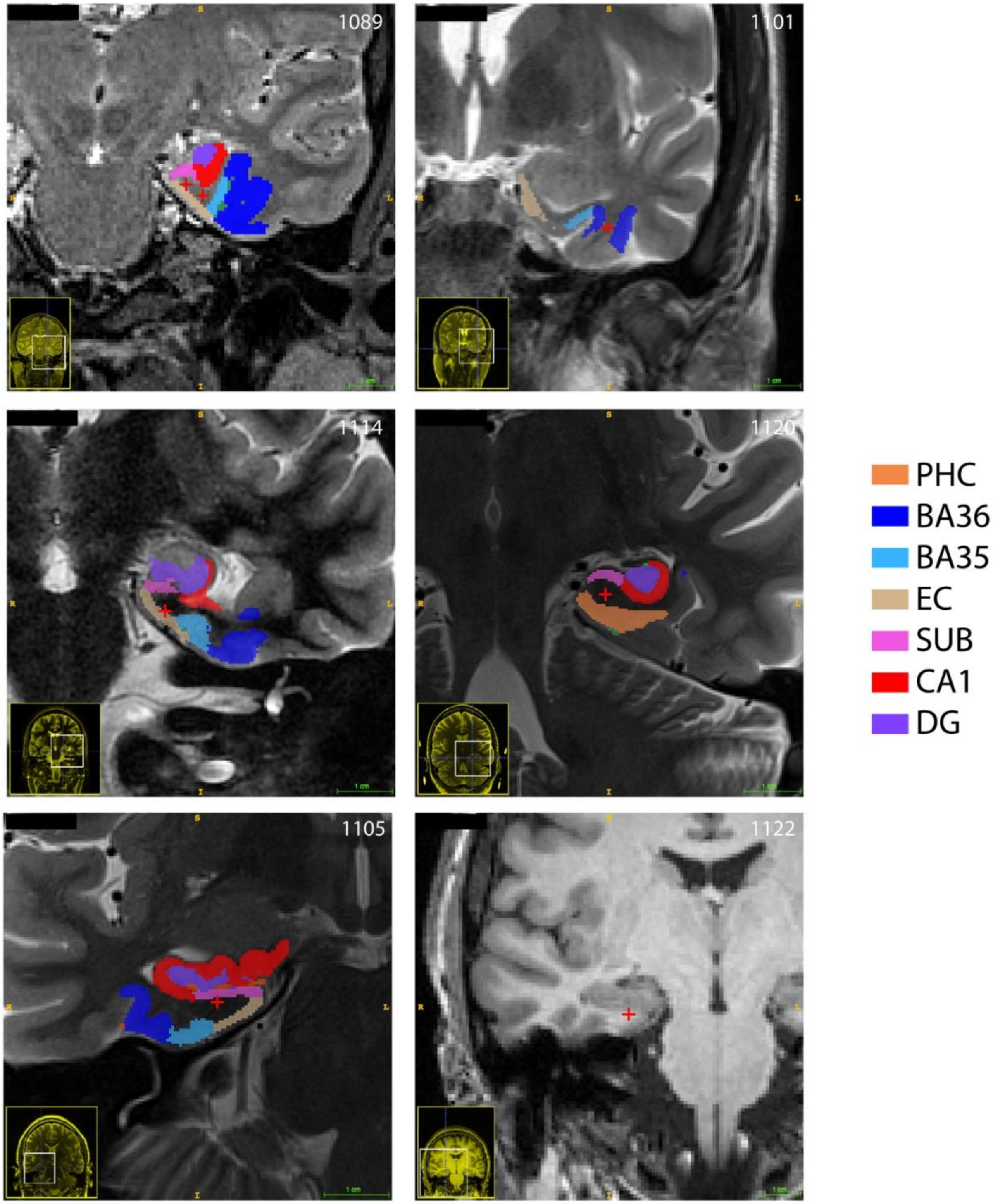

Supplementary Figure 1. MRI and electrode placements in white matter. In 6 subjects (7 distinct stimulation sites), bipolar stimulation electrode midpoints fell directly in MTL white matter, indicated by red crosses at the midpoint of the anode/cathode contacts. Overlaid segmentations show MTL subregions, according to the color legend above (no segmentation available for subject 1122). PHC, parahippocampal cortex; BA36, Broadmann area 36; BA35, Broadmann area 35/perirhinal cortex; EC, entorhinal cortex; SUB, subiculum; DG, dentate gyrus.

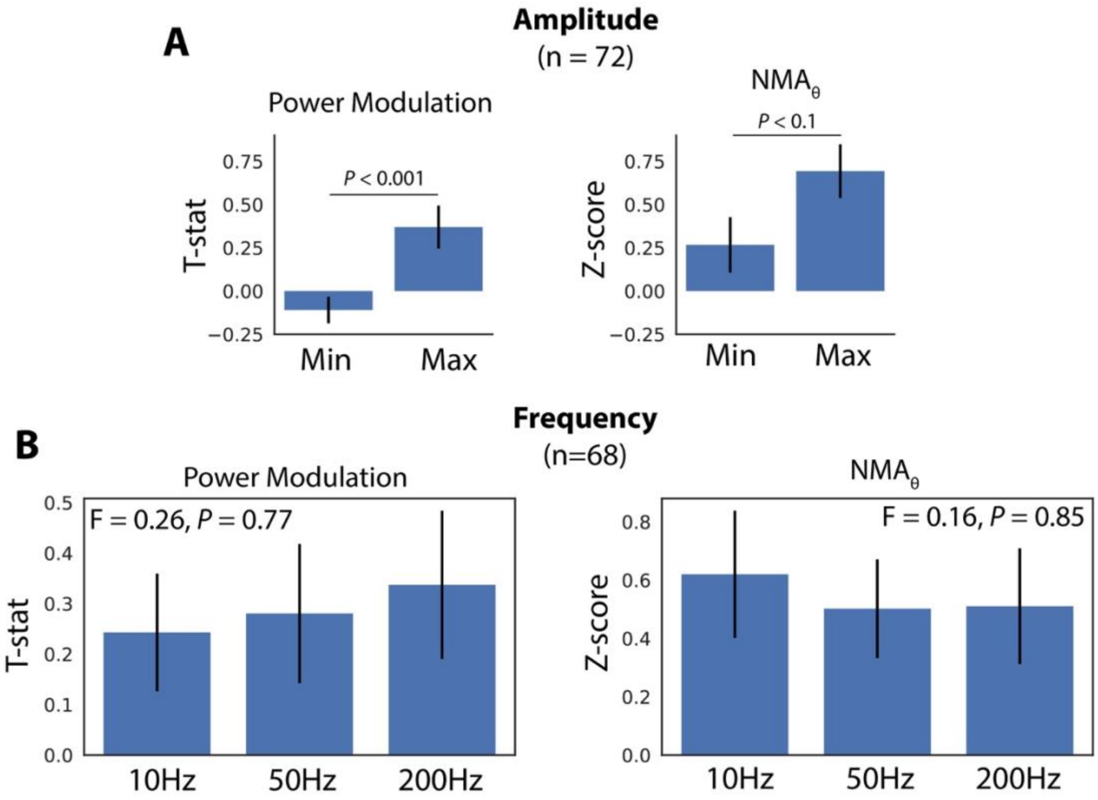

Supplementary Figure 2. Analysis of stimulation parameters on evoked power and theta network-mediated activation (NMA$_\theta$). For each stimulation site, stimulation parameters were varied across amplitudes (three amplitudes, typically between 0.5-2 mA, 0.25 mA apart) and frequencies (10, 25, 50, 100, 200 Hz; see Methods for details). **(A)** The average theta (5-8 Hz) power evoked by stimulation (measured as the average pre-vs.-post T-statistic across the top 5 most strongly-connected electrodes to the stimulation target) is sensitive to stimulation amplitude, comparing the evoked power with the minimum delivered amplitude versus the maximum amplitude at each stimulation site (paired T-test, $P <$ 0.001). The theta network-mediated activation (NMA$_\theta$; see Figure 3 and Methods for details) is marginally sensitive to amplitude ($P < 0.1$). **(B)** Repeated measures ANOVA indicated no effect of sitmulation frequency (measured at 10 Hz, 50 Hz, 200 Hz) on evoked power or NMA$_\theta$. Error bars show +/- 1 SEM.

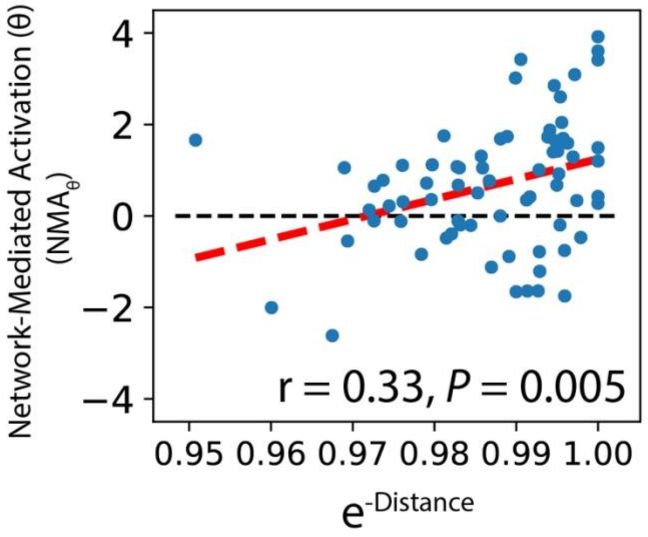

Supplementary Figure 3. Correlation of NMA_θ and distance to nearest white matter. There is a significant linear relationship (r = 0.33, *P* = 0.005) between an electrode's network-mediated activation in the theta band (NMA_θ) and distance to nearest white matter ($e^{-(dist)}$, where 1 indicates placement within white matter).

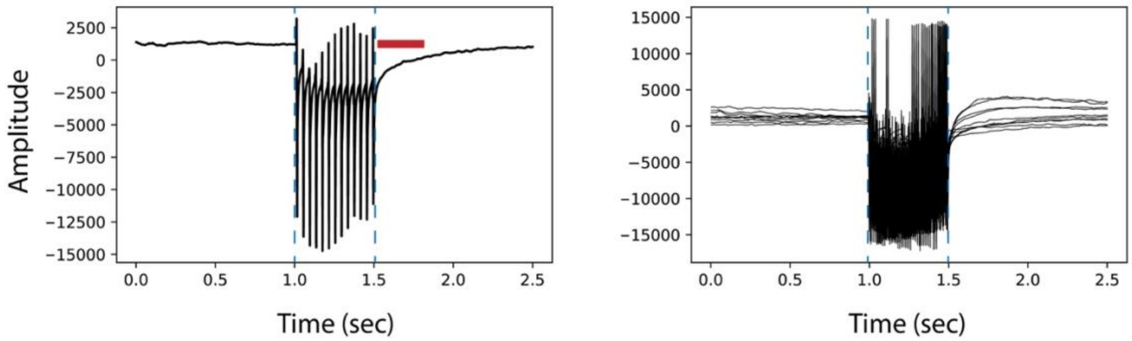

Supplementary Figure 4. Depiction of post-stimulation artifact. A subset of channels (average of 28% per subject) exhibited a non-physiologic post-stimulation artifact, characterized by a slowly decaying voltage offset immediately after the last stimulation pulse. A typical example of this artifact for one stimulation event, is shown on the left, and 10 representative examples are shown on the right, demonstrating their consistency across events. The red bar indicates the 350 ms post-stimulation period used to assess a channel for rejection (see Methods). Blue dashed lines indicate the 500 ms stimulation interval. For a representative artifact-free trace, see Figure 1E-F.
